# 4-Methyl-*N*-[(5-nitro­thio­phen-2-yl)methyl­idene]aniline

**DOI:** 10.1107/S1600536811030297

**Published:** 2011-08-02

**Authors:** Mingjian Cai, Xiuge Wang, Tao Sun

**Affiliations:** a Department of Chemistry, Tangshan Normal University, Tangshan 063000, People’s Republic of China

## Abstract

The title compound, C_12_H_10_N_2_O_2_S, is a Schiff base formed from *p*-toluidine and 5-nitro­thio­phene-2-carbaldehyde. The C=N bond adopts an *E* configuration. The benzene and thio­phene rings form a dihedral angle of 9.2 (1)°.

## Related literature

For the use of Schiff bases as polydentate ligands, see: Bourget-Merle *et al.*(2002 ▶); Halbach & Hamaker (2006 ▶); Meiswinkel & Werner (2004 ▶); Xiao *et al.* (2006 ▶); Lagadic (2006 ▶). For their biological activity, see: Siddiqui *et al.* (2006 ▶).
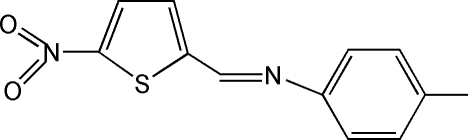


## Experimental

### 

#### Crystal data

C_12_H_10_N_2_O_2_S
*M* *_r_* = 246.28
Monoclinic, 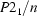

*a* = 4.7606 (4) Å
*b* = 22.415 (2) Å
*c* = 10.7008 (15) Å
β = 92.566 (13)°
*V* = 1140.7 (2) Å^3^
*Z* = 4
Mo *K*α radiation
μ = 0.27 mm^−1^
*T* = 113 K
0.20 × 0.18 × 0.12 mm

#### Data collection

Rigaku Saturn724 CCD diffractometer
Absorption correction: multi-scan (*CrystalClear*; Rigaku/MSC, 2002 ▶) *T*_min_ = 0.947, *T*_max_ = 0.968
14437 measured reflections
2699 independent reflections
2325 reflections with *I* > 2σ(*I*)
*R*_int_ = 0.043

#### Refinement

*R*[*F* ^2^ > 2σ(*F* ^2^)] = 0.040
*wR*(*F* ^2^) = 0.098
*S* = 1.09
2699 reflections
155 parameters
H-atom parameters constrained
Δρ_max_ = 0.30 e Å^−3^
Δρ_min_ = −0.27 e Å^−3^

### 

Data collection: *CrystalClear* (Rigaku/MSC, 2002 ▶); cell refinement: *CrystalClear*; data reduction: *CrystalClear*; program(s) used to solve structure: *SHELXS97* (Sheldrick, 2008 ▶); program(s) used to refine structure: *SHELXL97* (Sheldrick, 2008 ▶); molecular graphics: *DIAMOND* (Crystal Impact, 2009 ▶); software used to prepare material for publication: *CrystalStructure* (Rigaku/MSC, 2006 ▶).

## Supplementary Material

Crystal structure: contains datablock(s) global, I. DOI: 10.1107/S1600536811030297/ld2020sup1.cif

Structure factors: contains datablock(s) I. DOI: 10.1107/S1600536811030297/ld2020Isup2.hkl

Supplementary material file. DOI: 10.1107/S1600536811030297/ld2020Isup3.cml

Additional supplementary materials:  crystallographic information; 3D view; checkCIF report

## Figures and Tables

**Fig. 1.**
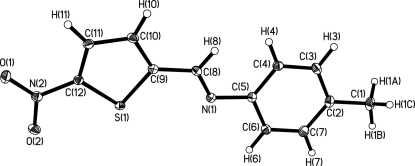
View of the molecule of (I) showing the atom-labelling scheme. Displacement ellipsoids are drawn at the 50% probability level.

## supplementary crystallographic information

### Comment

In recent years, heterocycle-containing Schiff bases have gained much attention as versatile polydentate ligands suitable for various metal chelations resulting in a variety of interesting coordination modes (Xiao *et al.*, 2006; Bourget-Merle *et al.*, 2002; Meiswinkel & Werner, 2004; Halbach & Hamaker, 2006; Lagadic, 2006). They also represent an important class of biologically active compounds (Siddiqui *et al.*, 2006). Herein, we report the synthesis and crystal structure of the title compound (I), a new heterocycle-containing Schiff base. The molecular structure of (I) is shown on Fig. 1. In the molecule of (I), the two aromatic benzene and thiophene rings form a dihedral angle of 9.2 (1)°. The deviation from planarity can be explained by steric repulsion between the phenyl ring and methylene group.

### Experimental

The solution of *p*-toluidine and 5-nitrothiophene-2-carbaldehyde in methanol was stirred for 10 h at ambient temperature. Then the crude product was isolated by filtration and recrystallized from methanol to yield yellowish title compound. Finally, the compound was dissolved in a small amount of acetone and the solution was kept for 3 days at ambient temperature to give rise to yellowish needle-like crystals by slowly evaporating the solvent.

### Refinement

All H atoms were positioned geometrically(C—H=0.93–0.98 Å),and refined as riding with *U*_iso_(H)=1.2*U*_eq_ of the adjacent carbon atom (1.5*U*_eq_ for methyl hydrogens). The positions of methyl hydrogens were rotationally optimized (AFIX 137).

### Crystal data

**Table:**

C_12_H_10_N_2_O_2_S	*F*(000) = 512
*M**_r_* = 246.28	*D*_x_ = 1.434 Mg m^−^^3^
Monoclinic, *P*2_1_/*n*	Mo *K*α radiation, λ = 0.71073 Å
*a* = 4.7606 (4) Å	Cell parameters from 4173 reflections
*b* = 22.415 (2) Å	θ = 1.8–27.9°
*c* = 10.7008 (15) Å	µ = 0.27 mm^−^^1^
β = 92.566 (13)°	*T* = 113 K
*V* = 1140.7 (2) Å^3^	Prism, colorless
*Z* = 4	0.20 × 0.18 × 0.12 mm

### Data collection

**Table:**

Rigaku Saturn724 CCD diffractometer	2699 independent reflections
Radiation source: rotating anode	2325 reflections with *I* > 2σ(*I*)
multilayer	*R*_int_ = 0.043
Detector resolution: 14.22 pixels mm^-1^	θ_max_ = 27.9°, θ_min_ = 1.8°
ω and φ scans	*h* = −6→6
Absorption correction: multi-scan (*CrystalClear*; Rigaku/MSC, 2002)	*k* = −29→29
*T*_min_ = 0.947, *T*_max_ = 0.968	*l* = −13→14
14437 measured reflections	

### Refinement

**Table:**

Refinement on *F*^2^	Primary atom site location: structure-invariant direct methods
Least-squares matrix: full	Secondary atom site location: difference Fourier map
*R*[*F*^2^ > 2σ(*F*^2^)] = 0.040	Hydrogen site location: inferred from neighbouring sites
*wR*(*F*^2^) = 0.098	H-atom parameters constrained
*S* = 1.09	*w* = 1/[σ^2^(*F*_o_^2^) + (0.0505*P*)^2^ + 0.1298*P*] where *P* = (*F*_o_^2^ + 2*F*_c_^2^)/3
2699 reflections	(Δ/σ)_max_ < 0.001
155 parameters	Δρ_max_ = 0.30 e Å^−^^3^
0 restraints	Δρ_min_ = −0.27 e Å^−^^3^

### Special details

**Table:**

Experimental. Rigaku *CrystalClear*-SM Expert 2.0 r2
Geometry. All e.s.d.'s (except the e.s.d. in the dihedral angle between two l.s. planes) are estimated using the full covariance matrix. The cell e.s.d.'s are taken into account individually in the estimation of e.s.d.'s in distances, angles and torsion angles; correlations between e.s.d.'s in cell parameters are only used when they are defined by crystal symmetry. An approximate (isotropic) treatment of cell e.s.d.'s is used for estimating e.s.d.'s involving l.s. planes.
Refinement. Refinement of *F*^2^ against ALL reflections. The weighted *R*-factor *wR* and goodness of fit *S* are based on *F*^2^, conventional *R*-factors *R* are based on *F*, with *F* set to zero for negative *F*^2^. The threshold expression of *F*^2^ > σ(*F*^2^) is used only for calculating *R*-factors(gt) *etc*. and is not relevant to the choice of reflections for refinement. *R*-factors based on *F*^2^ are statistically about twice as large as those based on *F*, and *R*- factors based on ALL data will be even larger.

### Fractional atomic coordinates and isotropic or equivalent isotropic displacement parameters (Å^2^)

**Table:**

	*x*	*y*	*z*	*U*_iso_*/*U*_eq_	
S1	0.12948 (8)	0.223694 (17)	0.24141 (3)	0.01725 (12)	
O1	−0.4301 (2)	0.10562 (5)	0.14443 (11)	0.0279 (3)	
O2	−0.2638 (2)	0.13512 (5)	0.32720 (10)	0.0257 (3)	
N1	0.5743 (3)	0.32247 (6)	0.21001 (11)	0.0170 (3)	
N2	−0.2748 (3)	0.13695 (6)	0.21144 (12)	0.0200 (3)	
C1	1.3858 (3)	0.51381 (7)	0.21589 (19)	0.0294 (4)	
H1A	1.5510	0.5020	0.1704	0.044*	
H1B	1.4432	0.5230	0.3028	0.044*	
H1C	1.2998	0.5492	0.1763	0.044*	
C2	1.1750 (3)	0.46324 (7)	0.21303 (16)	0.0220 (3)	
C3	1.0879 (3)	0.43701 (7)	0.10002 (15)	0.0223 (3)	
H3	1.1645	0.4507	0.0248	0.027*	
C4	0.8909 (3)	0.39115 (7)	0.09487 (14)	0.0197 (3)	
H4	0.8337	0.3742	0.0164	0.024*	
C5	0.7759 (3)	0.36966 (7)	0.20461 (14)	0.0169 (3)	
C6	0.8680 (3)	0.39494 (7)	0.31778 (14)	0.0199 (3)	
H6	0.7965	0.3804	0.3935	0.024*	
C7	1.0628 (3)	0.44113 (7)	0.32203 (16)	0.0233 (4)	
H7	1.1207	0.4579	0.4005	0.028*	
C8	0.4454 (3)	0.30556 (7)	0.10888 (14)	0.0189 (3)	
H8	0.4859	0.3248	0.0327	0.023*	
C9	0.2385 (3)	0.25772 (7)	0.10682 (14)	0.0173 (3)	
C10	0.1041 (3)	0.23474 (7)	0.00118 (14)	0.0208 (3)	
H10	0.1382	0.2483	−0.0809	0.025*	
C11	−0.0895 (3)	0.18913 (7)	0.02644 (14)	0.0194 (3)	
H11	−0.2003	0.1683	−0.0354	0.023*	
C12	−0.0955 (3)	0.17906 (7)	0.15201 (14)	0.0166 (3)	

### Atomic displacement parameters (Å^2^)

**Table:**

	*U*^11^	*U*^22^	*U*^33^	*U*^12^	*U*^13^	*U*^23^
S1	0.0186 (2)	0.0195 (2)	0.01358 (19)	0.00044 (15)	−0.00004 (15)	−0.00052 (14)
O1	0.0285 (6)	0.0275 (6)	0.0275 (6)	−0.0102 (5)	−0.0009 (5)	−0.0022 (5)
O2	0.0313 (7)	0.0288 (6)	0.0174 (6)	−0.0008 (5)	0.0046 (5)	0.0029 (5)
N1	0.0166 (6)	0.0168 (6)	0.0174 (6)	0.0015 (5)	0.0004 (5)	0.0009 (5)
N2	0.0205 (7)	0.0200 (7)	0.0195 (7)	0.0020 (5)	0.0019 (5)	0.0003 (5)
C1	0.0210 (8)	0.0204 (8)	0.0472 (11)	−0.0026 (7)	0.0047 (8)	0.0001 (8)
C2	0.0153 (7)	0.0165 (8)	0.0345 (9)	0.0031 (6)	0.0018 (7)	0.0011 (7)
C3	0.0198 (8)	0.0211 (8)	0.0264 (8)	0.0025 (6)	0.0061 (7)	0.0057 (7)
C4	0.0202 (8)	0.0203 (8)	0.0187 (8)	0.0022 (6)	0.0008 (6)	−0.0005 (6)
C5	0.0134 (7)	0.0162 (7)	0.0210 (8)	0.0022 (6)	0.0002 (6)	0.0009 (6)
C6	0.0191 (7)	0.0218 (8)	0.0187 (7)	−0.0004 (6)	−0.0004 (6)	0.0023 (6)
C7	0.0222 (8)	0.0230 (8)	0.0245 (8)	−0.0011 (7)	−0.0025 (7)	−0.0018 (7)
C8	0.0194 (7)	0.0206 (8)	0.0168 (7)	0.0007 (6)	0.0019 (6)	0.0025 (6)
C9	0.0172 (7)	0.0189 (7)	0.0158 (7)	0.0018 (6)	0.0013 (6)	0.0005 (6)
C10	0.0211 (8)	0.0272 (8)	0.0142 (7)	−0.0010 (7)	0.0004 (6)	0.0013 (6)
C11	0.0185 (7)	0.0224 (8)	0.0173 (7)	−0.0005 (6)	0.0007 (6)	−0.0032 (6)
C12	0.0157 (7)	0.0168 (7)	0.0173 (7)	0.0007 (6)	0.0013 (6)	−0.0016 (6)

### Geometric parameters (Å, °)

**Table:**

S1—C12	1.7237 (15)	C3—H3	0.9500
S1—C9	1.7298 (15)	C4—C5	1.403 (2)
O1—N2	1.2271 (16)	C4—H4	0.9500
O2—N2	1.2382 (16)	C5—C6	1.390 (2)
N1—C8	1.277 (2)	C6—C7	1.389 (2)
N1—C5	1.4312 (19)	C6—H6	0.9500
N2—C12	1.4398 (19)	C7—H7	0.9500
C1—C2	1.513 (2)	C8—C9	1.456 (2)
C1—H1A	0.9800	C8—H8	0.9500
C1—H1B	0.9800	C9—C10	1.374 (2)
C1—H1C	0.9800	C10—C11	1.411 (2)
C2—C3	1.391 (2)	C10—H10	0.9500
C2—C7	1.395 (2)	C11—C12	1.364 (2)
C3—C4	1.391 (2)	C11—H11	0.9500
			
C12—S1—C9	89.77 (7)	C4—C5—N1	125.10 (13)
C8—N1—C5	118.86 (13)	C7—C6—C5	121.08 (15)
O1—N2—O2	124.27 (13)	C7—C6—H6	119.5
O1—N2—C12	118.08 (13)	C5—C6—H6	119.5
O2—N2—C12	117.65 (13)	C6—C7—C2	121.15 (15)
C2—C1—H1A	109.5	C6—C7—H7	119.4
C2—C1—H1B	109.5	C2—C7—H7	119.4
H1A—C1—H1B	109.5	N1—C8—C9	122.04 (14)
C2—C1—H1C	109.5	N1—C8—H8	119.0
H1A—C1—H1C	109.5	C9—C8—H8	119.0
H1B—C1—H1C	109.5	C10—C9—C8	125.35 (14)
C3—C2—C7	117.78 (14)	C10—C9—S1	111.94 (12)
C3—C2—C1	120.39 (15)	C8—C9—S1	122.70 (11)
C7—C2—C1	121.84 (15)	C9—C10—C11	113.47 (14)
C4—C3—C2	121.40 (15)	C9—C10—H10	123.3
C4—C3—H3	119.3	C11—C10—H10	123.3
C2—C3—H3	119.3	C12—C11—C10	110.57 (14)
C3—C4—C5	120.55 (14)	C12—C11—H11	124.7
C3—C4—H4	119.7	C10—C11—H11	124.7
C5—C4—H4	119.7	C11—C12—N2	125.62 (14)
C6—C5—C4	118.01 (14)	C11—C12—S1	114.25 (12)
C6—C5—N1	116.87 (13)	N2—C12—S1	120.10 (11)
			
C7—C2—C3—C4	1.4 (2)	N1—C8—C9—S1	5.1 (2)
C1—C2—C3—C4	−178.92 (14)	C12—S1—C9—C10	0.04 (12)
C2—C3—C4—C5	−0.6 (2)	C12—S1—C9—C8	179.15 (13)
C3—C4—C5—C6	−0.9 (2)	C8—C9—C10—C11	−179.16 (14)
C3—C4—C5—N1	−179.45 (13)	S1—C9—C10—C11	−0.08 (17)
C8—N1—C5—C6	167.13 (14)	C9—C10—C11—C12	0.09 (19)
C8—N1—C5—C4	−14.3 (2)	C10—C11—C12—N2	178.01 (13)
C4—C5—C6—C7	1.6 (2)	C10—C11—C12—S1	−0.06 (17)
N1—C5—C6—C7	−179.80 (14)	O1—N2—C12—C11	2.7 (2)
C5—C6—C7—C2	−0.7 (2)	O2—N2—C12—C11	−176.72 (14)
C3—C2—C7—C6	−0.8 (2)	O1—N2—C12—S1	−179.35 (11)
C1—C2—C7—C6	179.55 (14)	O2—N2—C12—S1	1.25 (18)
C5—N1—C8—C9	179.73 (13)	C9—S1—C12—C11	0.01 (12)
N1—C8—C9—C10	−175.90 (15)	C9—S1—C12—N2	−178.17 (12)
